# Building a framework for inclusion in health services research: Development of and pre-implementation faculty and staff attitudes toward the Diversity, Equity, and Inclusion (DEI) plan at Mayo Clinic

**DOI:** 10.1017/cts.2020.575

**Published:** 2021-01-05

**Authors:** Felicity T. Enders, Elizabeth H. Golembiewski, Laura M. Pacheco-Spann, Megan Allyse, Michelle M. Mielke, Joyce E. Balls-Berry

**Affiliations:** 1 Division of Biostatistics, Department of Health Sciences Research, Mayo Clinic, Rochester, MN, USA; 2 Division of Health Care Policy and Research, Department of Health Sciences Research, Mayo Clinic, Rochester, MN, USA; 3 Department of Health Sciences Research, Mayo Clinic, Jacksonville, FL, USA; 4 Division of Epidemiology, Department of Health Sciences Research, Mayo Clinic, Rochester, MN, USA; 5 Department of Neurology, Mayo Clinic, Rochester, MN, USA; 6 Department of Neurology, Washington University, St. Louis, MO, USA

**Keywords:** Diversity, equity, inclusion, organizational culture, academic medicine

## Abstract

**Objective:**

To mitigate the impact of racism, sexism, and other systemic biases, it is essential for organizations to develop strategies to address their diversity, equity and inclusion (DEI) climates. The objective of this formative evaluation was to assess Mayo Clinic Department of Health Sciences Research (HSR) faculty and staff perceptions toward a proposed departmental DEI plan and to explore findings by diversity and professional subgroups.

**Materials and methods:**

Key plan components include recruitment and support for diverse individuals; training for all HSR employees and leaders; and a review system to capture diversity and inclusion feedback for leaders. Additional activities include building inclusion “nudges” into existing performance reviews. To assess pre-implementation beliefs about specific plan components, we polled attendees at a departmental staff meeting in July 2020.

**Results:**

Overall, respondents (*n* = 162) commonly endorsed a blinded promotion review process and DEI training for all staff and leaders as most important. In contrast, respondents expressed less support for plan activities related to “nudges.” However, attitudes among certain diversity or professional groups toward specific plan activities diverged from their non-diversity group counterparts. Qualitative feedback indicated awareness of the need to address DEI issues.

**Discussion:**

Overall, HSR faculty and staff respondents conveyed support for the plan. However, some specific plan activities were perceived differently by members of certain diversity or professional subgroups.

**Conclusion:**

These findings present a DEI framework on which other institutions can build and point to future directions for how DEI activities may be differentially perceived by impacted faculty and staff.

## Introduction

Racism, sexism, and other forms of systemic bias and discrimination are pervasive in the US society, and this is reflected in academic medical institutions. Decades of research has established that systems of bias are inextricably linked to human health as well as health care access and utilization [1,2], underscoring the importance of a diverse workforce and inclusive organizational cultures among institutions associated with health care delivery and research. However, although the health services research workforce in the USA represents a multidisciplinary community of scholars with an expressed commitment to furthering equity in health care [3], many identities remain underrepresented in the ranks of academic medical centers and research institutions [4,5]. For example, less than 5% of all US medical school faculty are Black or Latinx, and only 35.3% are women [6]. Beyond representation, serious disparities in funding, career satisfaction, formal compensation, and other opportunities persist [7,8]. Black investigators are 10% points less likely than White applicants to be awarded NIH research funding, even after controlling for educational background, research productivity, and employer characteristics [9]. Female scientists, who comprise over half of health services researchers [10], are paid considerably less than their male counterparts, on par with national estimates of gender disparity in pay levels [11]. For members of these and other historically marginalized identity groups, such as LGBTQ+ or nonbinary individuals, foreign nationals, and persons with physical disabilities or mental health conditions, the need for change is clear [12].

In a decentralized field like health services research that spans many disciplines, efforts to improve culture at an *organizational* level are extremely important [13]. An organization’s diversity and inclusion climate may be influenced by several factors, including the role modeling of its leaders, its mission statements and goals, the programs and issues that the organization invests in and prioritizes, the formal and informal rules and policies that may unintentionally disadvantage certain groups, and the individual biases and attitudes of members of the organization. Such individual biases and attitudes are likely influenced, consciously or unconsciously, by power differentials inherent either in society or within the organization’s culture, such as male vs. female or senior vs. junior faculty. All of these factors contribute to the psychological safety of individuals within the organization, that is, the degree to which individuals feel that they can be themselves and be welcomed and valued, without fear of discrimination. In turn, people who feel that their identities are safe and valued are more productive and happier in their jobs [14].

To ensure that staff members can feel safe, valued, and productive, it is essential for organizations to develop strategies to evaluate and address the diversity and inclusion climate within their institution or division. At Mayo Clinic, a large academic medical center with faculty and staff employed in its Department of Health Sciences Research (HSR) across sites in Minnesota, Florida, and Arizona, a task force was formed in 2018 to develop a department-wide plan for diversity, equity, and inclusion (DEI) efforts. Consistent with the Association for Clinical and Translation Science (ACTS) strategic objective to develop a diverse and inclusive pipeline of future health services and translational science leaders [15], the Mayo Clinic HSR DEI plan is centered on increasing overall and subgroups’ sense of belonging and overall diversity within the institution. Of note, the HSR plan sits within the context of a larger Mayo Clinic institutional plan that leverages departmental leaders to make changes tailored to the needs of faculty and staff in individual divisions. The purpose of this paper is to detail the components of the Department of HSR DEI plan and report on pre-implementation HSR staff attitudes toward specific activities proposed within the plan.

## Materials and Methods

### Setting

The Department of HSR at Mayo Clinic is a multidisciplinary group of approximately 90 doctoral-level faculty and more than 400 staff (typically not doctoral-level) employees located across campuses in Rochester, MN, Phoenix, AZ, and Jacksonville, FL. In 2018, a seven-member diversity and inclusion task force was convened by the HSR department chair to develop a proposal for a formal departmental DEI program. Through a year-long process, the task force utilized anonymous free text comments from members of the Department of HSR on DEI issues they had faced. Based upon these comments and the research literature, the task force developed an initial multi-pronged approach that formed the basis of the plan presented here. The first step was to add a new leadership dyad [16] (i.e., faculty and staff leaders, respectively) within the department to fully develop and implement the plan. In 2019, the HSR department chair led a search committee for the new Associate Chair for DEI (faculty leader) and the new Allied Health Leader for DEI (staff leader). Following establishment of these individuals (Enders and Pacheco-Spann, respectively) in 2020, they were provided with salary and administrative assistant support and included in the department’s executive committee.

#### DEI Plan Components

The principal objectives of the DEI plan in the Department of HSR are twofold: 1) to increase overall and subgroups’ sense of belonging and 2) increase overall diversity. While many organizational approaches to increasing DEI in academia have been documented, many of these focus on interventions for specific subgroups – namely, female and underrepresented racial and ethnic minority faculty [17–19]. Mayo Clinic HSR DEI plan authors approached its development from a broader faculty “life course” perspective, attempting to identify critical junctures at which faculty and staff from a variety of diverse backgrounds are often hindered through biases in promotion and review processes, lack of appropriate mentorship, and majority cultures that may seem hostile to diverse individuals. In addition, plan authors drew from their own experiences as career academics from diverse backgrounds.

The DEI efforts described in the plan focus on groups that have historically been the subject of bias and/or discrimination (e.g., women, racial and ethnic minorities, foreign nationals, LGBTQ+ or nonbinary individuals, those with disabilities or chronic health issues, and religious minorities), as well as groups specific to academic research settings and the Mayo Clinic organizational structure. These latter groups include research staff (e.g., bachelors- and masters-level statisticians, research coordinators, etc.), research temporary professionals (e.g., postdoctoral fellows, research trainees), staff members based outside of Mayo Clinic’s primary campus in Rochester (e.g., in Arizona or Florida), remote workers, contractors, and staff who do not hold leadership positions (e.g., as chair or supervisors) within the institution.

Note: In the interest of brevity, throughout this paper, we refer to members of these personally or professionally disadvantaged groups collectively as “diverse” or “minority” individuals, and their nondiverse counterparts as “majority” individuals.

**Fig. 1.**
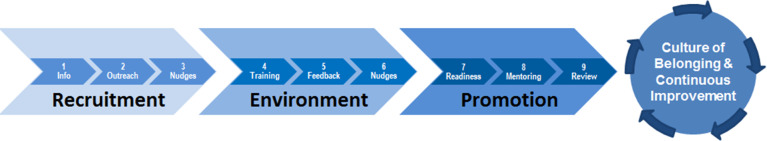
Mayo Clinic Health Sciences Research Diversity, Equity, and Inclusion plan overview and components.

The cornerstones of this work are 1) recruitment and support for diverse individuals within HSR (“Recruitment”); 2) training for all HSR employees and leaders (“Culture”); and 3) a Leadership 180 system to capture diversity and inclusion feedback for HSR leadership (“Promotion”; see Fig. 1). Additional complementary activities proposed in the plan include building inclusion “nudges” into existing performance reviews and annual assessments. Finally, we plan to survey all HSR faculty and staff every 2 years on sense of “belonging,” a positive measure of inclusion with important consequences for well-being [20].

### 1. Recruitment

In the Recruitment component, we will focus on increasing outreach to a diverse applicant pool for faculty and staff, as well as assessing diverse junior faculty and staff already employed at Mayo Clinic for their readiness for promotion to more senior positions within HSR. In addition, any individuals from diversity groups who have trouble integrating into Mayo Clinic culture or working with majority individuals at the institution will be eligible to receive coaching to help them better cope with challenging situations. We acknowledge that this places the onus of change on faculty and staff from diversity groups rather than on the institution or majority individuals themselves. However, we devised this approach with the awareness that societal and institutional changes are slow and many diverse individuals at Mayo Clinic and elsewhere may need help now in order to facilitate their career progression.

#### Outreach

We will focus on increasing outreach to a diverse faculty and staff applicant pool. We hope to achieve this through a mix of high cost/time (recruitment fairs, conference booths, talks at institutions) and low cost/time (targeted email lists, the use of social media) opportunities.

#### Readiness Assessment

Diverse faculty and staff who are already working at Mayo are ideal targets for increasing diversity in HSR’s tenured faculty. However, all aspiring senior faculty must both meet criteria for appointment and demonstrate a strong fit within Mayo culture. Nationally, diverse individuals are at greater risk for reduced productivity compared to their majority peers due to a variety of factors, including both cultural differences and psychological impact of an environment that may be perceived as unwelcoming. One mechanism for this may be the inaccessibility of certain “hidden curricula” of academic medical institutions such as Mayo Clinic, referring to embedded norms, values, and belief systems that may not be as readily apparent to individuals not part of the majority culture or background [21,22]. As such, in this component, we will assess diverse faculty and staff for their readiness for appointment to more senior positions in terms of both traditional metrics (e.g., productivity) as well as their cultural fit within the institution (e.g., are there individuals who need help accessing diversity-related hidden curricula?).

#### Coaching

Diverse individuals who are having issues integrating into Mayo Culture, or working with majority individuals at Mayo, will have the option to receive coaching to help them better integrate and cope with challenging situations. If needed, additional focused mentoring for increased productivity will also be provided.

### 2. Culture

Culture is a critical element of the plan, because it impacts all employees and is intended to increase the sense of belonging experienced by all HSR employees.

#### Training

We will start with training, including baseline training for all HSR employees as well as a training curriculum specific to leaders. The training for leaders will address topics likely to arise from the hierarchical nature of leadership, as those under a leader become more likely to perceive that individual as behaving like a bully [23]. The training will also introduce strategies for the prevention of problematic behaviors. Furthermore, research suggests that there are particular issues likely to arise for women in leadership that may be observed on an employee performance review, such as acting in a “masculine” manner [24]. Leaders need to be taught strategies for when to take this feedback back to the giver, and how, rather than including it directly on a performance review. Although the literature only addresses this for women, similar issues are likely to arise for other diversity groups.

#### Feedback

Feedback will be given to leaders through a novel Leadership 180 feedback system embedded within our HSR DEI survey. In the Leadership 180 system, all individuals under a leader will provide quantitative (single-item Likert) and optional qualitative (start/stop/keep) responses to be given to that leader. The Leadership 180 will include a reminder that women and racial or ethnic minority leaders tend to receive nonspecific critical comments, so specific comments that might be given to any leader are preferred. Comments will likely be scrubbed by DEI leadership prior to sharing with leaders. The format of feedback to the leader is a point of discussion in this report; most likely the leader will provide a self-reported assessment, privately review the responses (and their relation to the average for all HSR leaders), and privately reflect on potential areas of improvement. The Leadership 180 will be repeated every 2 years with the HSR DEI survey, and the survey will use the time frame of the prior 12 months to allow a leader time to modify behavior.

#### Nudges

Finally, within the Culture component of the plan, we will also add inclusion nudges. Research on diversity and inclusion shows that while training is essential, the knowledge it provides is a poor marker of behavior change [25]. Instead, providing “nudges” to appropriate behavior at the moment the behavior occurs is more likely to achieve our goals [26]. The concept of “nudging” stems from behavioral economics and involves the use of subtle prompts or shifts in design that enable or discourage a given behavior. Nudging has been widely applied in many different contexts, including diversity and inclusion, where it may serve to raise awareness of unconscious biases or even change organizational processes [27]. Examples of diversity and inclusion nudges include the pointed blinding of demographic information when assigning applications to hiring managers, or showing committees examples of gendered or racist language before asking them to review applications for promotion of current employees.

To that end, we plan to incorporate nudges into existing HSR faculty and staff performance review mechanisms. First, a framing nudge will be built into the 360 review process, a periodic review that solicits feedback about an employee from their supervisors, colleagues, and direct reports. Individuals completing and reviewing 360 review comments for another employee will be reminded of common biases that occur in feedback for women, racial and ethnic minorities, and other groups. We further plan a process nudge within the Mayo Clinic annual review process, in which individuals will be asked to reflect and plan how they will increase others’ sense of belonging over the next year. Other planned nudges include framing within the Leadership 180 and the process of blinded review for promotion.

### 3. Promotion

In the Promotion component, diverse individuals will be assessed for readiness with regard to their planned path to promotion. Those who identify a need will be offered mentoring and/or coaching to manage working relationships or gain tips to present themselves in a manner associated with success. Finally, a group within HSR will carry out a blinded review of all junior staff using metrics to assess readiness for promotion. For each doctoral-level staff member who is not yet at the highest rank, a one-page metrics assessment will be created showing key criteria used for promotion decisions (e.g., publications, grants, teaching, etc.). Rather than showing actual metric values, ranges will be utilized to help anonymize data. A team of experts will then evaluate proposed rank (without knowing true current rank). Results will be sent to the leader over the staff member as a mechanism to provide input and level the playing field across leaders who otherwise may have different unwritten criteria regarding readiness for promotion.

#### Assessment of DEI Plan Perceptions

In July 2020, the draft plan was presented to the DEI advisory board, a group of faculty and staff comprising faculty and staff with particular diversity characteristics to ensure that different perspectives are included in the plan. The advisory board provided feedback and made suggestions for the inclusion of additional diversity groups: parents with children at home, employees with a mental health condition, and employees who are overweight or obese. Next, the DEI plan was presented to HSR faculty and staff at an all-staff meeting. We obtained anonymous feedback from meeting attendees using Poll Everywhere, a real-time polling software for presentations [20]. Data collection for this report was deemed a quality improvement activity by the Mayo Clinic Institutional Review Board.

Respondents were instructed to select up to three activities from a list of DEI plan activities in response to each of the following questions: “Which 3 DEI activities are most important?”, “Which 3 DEI activities will make the most difference in HSR culture?”, and “Which 3 DEI activities would you suggest not doing?” The response choices included key activities from the DEI plan: training all HSR employees on DEI topics; training leaders on DEI leadership topics; assessment of “belonging” by diversity group; Leadership 180 feedback to leaders; annual review inclusion nudge; other inclusion nudges (360 review, search committee, outlook, etc.); recruitment; and blinded promotion review.

In addition, we asked poll respondents to anonymously indicate their membership in any or multiple diversity and professional groups. From a list of diversity groups, respondents were asked to select all that apply for each of the following: woman or female-presenting; a racial or ethnic minority underrepresented in academia (including Black, Hispanic/Latinx, Native American, or Pacific Islander); any other racial or ethnic minority group (such as Asian, Indian, or Middle Eastern); not the US citizen; speaks English as a second language; non-Christian identifying (because Mayo is affiliated with a Catholic entity the institution prioritizes Christian holidays); LGBTQ+; has a mental health condition; overweight or obese; or a parent with children at home. To assess professional diversity group membership, we asked attendees to select all that apply for each of the following: work division(s) within HSR; current job category (research temporary professional, faculty, or staff); whether they currently serve in a leadership position within HSR (e.g., chair, supervisor); and whether they are based outside of the Mayo Clinic Rochester downtown campus (i.e., full-time remote workers, or employees at Arizona or Florida sites). These diversity group options were not exhaustive and chosen in consultation with the DEI advisory board to elicit known perspectives of the HSR workforce.

Finally, we asked attendees to respond to three open-ended questions: “What does DEI mean to you?”, “How will you speak up on DEI issues you encounter?”, and “What will you do to make HSR more inclusive?”

### Analysis

We computed descriptive statistics from faculty and staff self-reported poll data on plan perceptions, diversity group memberships, and professional categories. Specifically, each variable was dichotomized as a yes/no response depending on whether a poll participant endorsed a given plan activity, diversity group, or professional category. We also ran bivariate comparisons to assess differences in DEI plan perceptions by each diversity or professional group and their corresponding non-diversity group, and we tested the comparisons statistically using chi-square or Fisher’s exact tests. Categories with fewer than 10 respondents are either not reported or were pooled for bivariate analyses. For example, “under-represented racial or ethnic minority” and “any other racial or ethnic minority group (such as Asian, Indian, or Middle Eastern)” were combined into a single variable representing all non-White respondents. Pre-specified comparisons were run for each diversity group vs. its non-diversity group for activities endorsed as most important, activities that will make the biggest impact on HSR culture, and activities suggested not doing, respectively. In these analyses, we intentionally did not adjust for testing of multiple relationships given the exploratory nature of this work, which we hope to use for future, more targeted research efforts. In addition, we reviewed qualitative responses to the three open-ended poll questions for common themes and suggestions.

Results were considered statistically significant at the *P* ≤ 0.05 level. All quantitative analyses were performed using JMP statistical software (version 4.0.3; SAS Institute, Cary, NC).

## Results

### Respondent Characteristics

A total of 162 HSR faculty and staff attendees responded to at least one question within the meeting poll, from a total of 312 meeting attendees (response rate: 51.9%). Nearly 42.6% (*n* = 69) reported having children at home and 60% of respondents (*n* = 96; 59.3%) identified as women. Only 9 (5.6%) identified as an underrepresented racial or ethnic minority, while 27 (16.7%) identified as an “other” racial or ethnic minority from groups not underrepresented in academia (i.e., Asian, Indian, or Middle Eastern). Speaking English as a second language was selected by 16.0% of respondents (*n* = 26). Approximately one-quarter of meeting attendees described themselves as overweight or obese (*n* = 41; 25.3%), a religious minority (*n* = 36; 22.2%), or as having a mental health condition (*n* = 37; 22.8%), respectively. Most attendees were employed as staff (*n* = 109; 67.3%). A minority of respondents (*n* = 22; 13.6%) reported holding one or multiple leadership positions. Over one-quarter of respondents (*n* = 45; 27.8%) were based outside of Rochester, MN at Mayo Clinic sites in Florida or Arizona, satellite locations, or as remote workers. See Table 1 for respondent characteristics.

**Table 1.** Respondent professional and personal characteristics (*n* = 162)

Division	*n*	%
Biomedical Statistics and Informatics	112	69.1
Epidemiology	15	9.3
Health Care Policy and Research	11	6.8
Digital Health Sciences	8	4.9
Job category		
Staff	109	67.3
Faculty	29	17.9
Research temporary professionals	5	3.1
Other professional characteristics		
Not in Rochester downtown (sites, teleworkers, 41st street)	45	27.8
Leadership	22	13.6
Personal characteristics		
Woman (female-presenting)	96	59.3
Parent with children at home	69	42.6
Overweight or obese	41	25.3
Mental health condition	37	22.8
Religious minority (not Christian)	36	22.2
Non underrepresented racial/ethnic minority (Asian, Indian, Middle Eastern)	27	16.7
English as a second language	26	16.0
Non-US citizen	23	14.2
Under-represented racial/ethnic minority (Black, Hispanic/Latinx, Native American, Pacific Islander)	9	5.6
LGBTQ+	5	3.1

### Perceptions of DEI Plan Activities

**Fig. 2.**
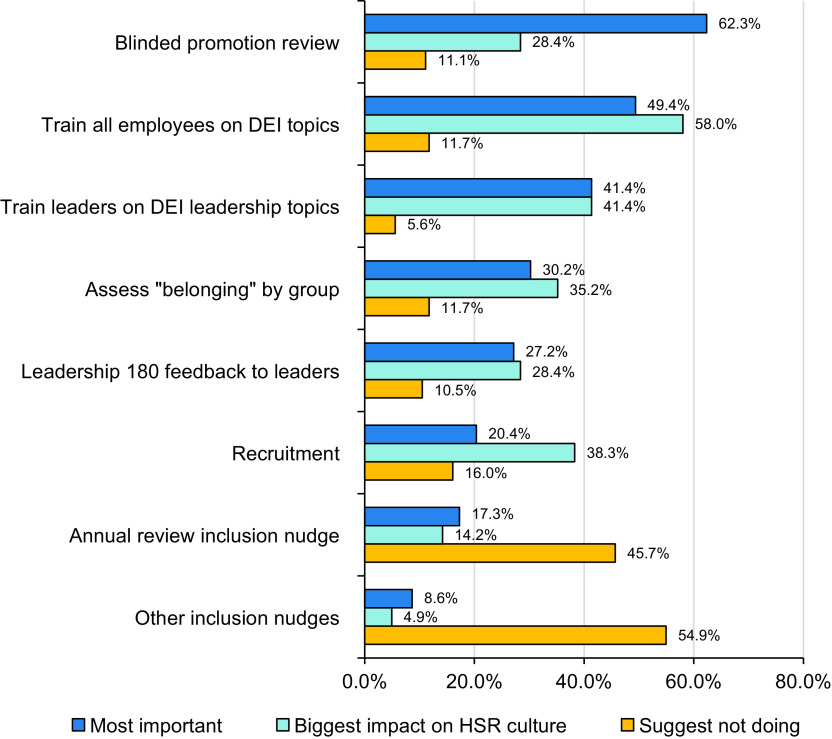
Perceptions of Diversity, Equity, and Inclusion (DEI) plan activities among Department of Health Sciences Research (HSR) staff meeting participants. Notes: Values represent percentage of all respondents (*n* = 162) endorsing a given activity for each question. Respondents were instructed to select up to three activities from the list in response to each of the following questions: “Which 3 DEI activities are most important?” (“Most important”), “Which 3 DEI activities will make the most difference in HSR culture?” (“Biggest impact on HSR culture”), and “Which 3 DEI activities would you suggest not doing?” (“Suggest not doing”).

Among all respondents, endorsements for “most important” DEI plan activities were for blinded promotion review (62.3%), training for all employees on DEI topics (49.4%), and training leaders on DEI topics specific to leadership (41.4%). Both DEI training for all employees (58.0%) and training for leaders on DEI leadership topics (41.4%) were endorsed as most potentially impactful on HSR culture, followed by recruitment (38.3%). Finally, in response to the question of which three activities should *not* be part of the DEI plan, respondents selected inclusion nudges (54.9%) and annual review inclusion nudge (45.7%). Approximately one-quarter of staff (26.0%) did not select any of the listed DEI activities in response to the question of which DEI activities they suggested not be done. See Fig. 2.

#### Diversity Groups

See Table 2, which presents bivariate comparisons for activities rated as “most important” by select diversity groups. A greater proportion of women (60.2%) than non-women (41.5%) endorsed training for all HSR staff (*P* = 0.04) as a “most important” plan activity. However, compared to participants who did not identify as a religious minority, a smaller proportion of religious minority respondents selected training for all HSR staff as a “most important” activity (42.9% of religious minorities vs. 58.6% of non-religious minority respondents; *P* = 0.11). Compared to respondents who did not identify as a racial or ethnic minority, a higher proportion of racial and ethnic minority respondents endorsed “other” inclusion nudges as one of the most important DEI activities (19.4% of minority respondents vs. 5.9% of non-minority respondents, *P* = 0.03). Among parents with children at home, the annual review inclusion nudge was endorsed as a “most important” activity (25.4% of parents vs. 9.2% of non-parents; *P* = 0.01). Finally, compared to native English speakers, a significantly smaller proportion of non-native English speakers chose the Leadership 180 feedback system as “most important” (13.0% of non-native speakers vs. 34.8% of native English speakers; *P* = 0.03).

**Table 2.** “Most important” DEI plan activities endorsed by select diversity vs. non-diversity groups

	Woman	Not a woman	Racial or ethnic minority	Not a racial or ethnic minority	Non-native English speaker	Native English speaker	Parent with children at home	Not a parent with children at home	Religious minority	Not a religious minority
n	93	40	36	102	23	109	68	66	36	99
Blinded promotion review	62	31	22	71	17	76	48	45	25	68
	(66.7%)	(77.5%)	(71.0%)	(59.6%)	(73.9%)	(69.0%)	(71.6%)	(68.2%)	(75.1%)	(69.4%)
Train all employees on DEI topics	56	17	17	56	13	60	38	35	15	58
	(60.2%)*	(41.5%)	(54.8%)	(54.4%)	(54.2%)	(54.6%)	(55.9%)	(53.0%)	(42.9%)	(58.6%)
Train leaders on DEI leadership topics	41	18	14	45	13	46	30	29	17	42
	(44.1%)	(43.9%)	(45.2%)	(43.7%)	(54.2%)	(41.8%)	(44.1%)	(43.9%)	(48.6%)	(42.4%)
Leadership 180 feedback to leaders	26	16	6	46	3	39	23	19	9	33
	(28.3%)	(40.0%)	(19.4%)	(35.6%)	(13.0%)*	(35.8%)	(34.3%)	(29.2%)	(26.5%)	(33.7%)
Annual review inclusion nudge	16	7	7	16	4	19	17	6	7	16
	(17.4%)	(17.5%)	(22.6%)	(15.8%)	(17.4%)	(17.4%)	(25.4%)*	(9.2%)	(20.6%)	(16.3%)
Other inclusion nudges	9	3	6	6	4	8	6	6	3	9
	(9.7%)	(7.5%)	(19.4%)*	(5.9%)	(17.4%)	(7.3%)	(9.0%)	(9.1%)	(8.6%)	(9.2%)

DEI, diversity, equity and inclusion.

Notes: Values and percentages refer to respondents within each group endorsing a given activity. Denominators for subgroup percentages vary slightly from the total N for a given subgroup due to differences in item response rates for diversity group membership. Differences between diversity groups and corresponding non-diversity groups were tested statistically using chi-square or Fisher’s exact tests (*P ≤ 0.05; **P ≤ 0.01; ***P ≤ 0.001). Respondents were instructed to select up to three activities from the list in response to the question: “Which 3 Diversity and Inclusion (DEI) plan activities are most important?” “Racial and ethnic minority” includes members of underrepresented racial and ethnic minority groups (Black, Hispanic/Latinx, American Indian or Alaska Native, Native Hawaiian or other Pacific Islander), and other non-White minority groups (Asian, Indian, or Middle Eastern). “Religious minority” refers to respondents who do not celebrate Christian holidays.

For activities endorsed as likely to make the biggest difference in HSR culture (data not shown), a significantly higher proportion of religious minorities (12.5%) vs. non-minority/Christian respondents (2.1%) selected other inclusion nudges as likely to make the biggest difference (*P* = 0.03). Parents of children at home were significantly more likely to endorse recruitment (57.8% of non-parents vs. 27.7% of parents; *P* < 0.001) and the annual review inclusion nudge (21.5% of parents vs. 3.9% of non-parents; *P* = 0.02) as activities that would make the biggest difference in HSR culture.

Finally, among DEI plan activities that respondent suggested not to do (data not shown), the Leadership 180 feedback system had a higher proportion of votes for “suggest not doing” among non-native English speakers (26.1% of non-native English speakers suggest not doing vs. 9.1% of native English speakers; *P* = 0.04) as well as racial and ethnicity minority respondents (27.6% of minority respondents suggest not doing vs. 7.3% of non-minority respondents; *P* = 0.005).

#### Professional Groups

See Table 3, which presents bivariate comparisons for activities rated as “most important” by professional group. Respondents in leadership positions were more likely to endorse the annual review inclusion nudge as most important (40.9% vs. 15.1% of non-leaders; *P* = 0.009). In addition, a greater proportion of staff (59.3%) than faculty (34.3%) rated training for all HSR staff as most important (*P* = 0.01). A greater proportion of leaders (30.0%) than non-leaders (11.8%) suggested not doing blinded promotion reviews (*P* = 0.05; data not shown).

**Table 3.** Diversity, Equity, and Inclusion (DEI) plan activities endorsed as a) most important and b) most impactful to departmental culture by professional subgroup

	Most important				Biggest impact on HSR culture			
	Not a leader	Leader	Staff	Faculty	Not a leader	Leader	Staff	Faculty
n	93	22	108	34	93	22	108	34
Blinded promotion review	83	13	71	25	38	7	35	10
	(69.2%)	(59.1%)	(65.7%)	(73.5%)	(32.2%)	(31.8%)	(33.0%)	(29.4%)
Train all employees on DEI topics	62	14	64	12	79	15	73	21
	(51.2%)	(63.6%)	(59.3%)**	(34.3%)	(66.9%)	(68.2%)	(68.9%)	(61.8%)
Train leaders on DEI leadership topics	56	9	48	17	58	8	54	12
	(46.3%)	(40.9%)	(44.4%)	(48.6%)	(49.6%)	(36.4%)	(50.9%)	(36.4%)
Leadership 180 feedback to leaders	37	5	30	12	37	7	34	10
	(31.1%)	(22.7%)	(28.0%)	(35.3%)	(31.4%)	(31.8%)	(32.1%)	(29.4%)
Annual review inclusion nudge	18**	9	18	9	37	7	34	10
	(15.1%)	(40.9%)	(16.8%)	(26.5%)	(31.4%)	(31.8%)	(32.1%)	(29.4%)
Other inclusion nudges	11	3	11	3	7	1	6	2
	(9.2%)	(13.6%)	(10.2%)	(8.8%)	(5.9%)	(4.6%)	(5.7%)	(5.9%)

Notes: Values and percentages refer to respondents within each group endorsing a given activity. Denominators for subgroup percentages vary slightly from the total N for a given subgroup due to differences in item response rates for professional group membership. Differences between professional groups were tested statistically using chi-square or Fisher’s exact tests (*P ≤ 0.05; **P ≤ 0.01; ***P ≤ 0.001). Respondents were instructed to select up to three activities from the list in response to the questions: “Which 3 DEI plan activities are most important?” and “Which 3 DEI activities will make the most difference in Department of Health Sciences Research (HSR) culture?” “Leadership” includes division heads, chairs, or supervisors.

### Qualitative Responses

Responses to open-ended questions, in general, indicated participant acknowledgment of existing DEI issues and endorsement of the need for DEI initiatives such as those proposed in the HSR plan. Respondents described what DEI looks like in practice from their perspective as well as specific individual and institutional actions needed to improve DEI climate within the department. A small number of participants expressed concerns about potential adverse effects of DEI efforts. Specific themes and representative quotes are presented in Table 4.

**Table 4.** Open-ended responses from all-staff meeting participants

Prompt	Theme	Representative quote(s)
What does DEI mean to you?	Representation	*Leaders like us* – *minorities, female, parents of young children, etc.… (F, white)*
		*Bringing new voices into the field. (M, white)*
	Mutual respect	*Treat everyone the way you wanted to be treated. Be respectful to everyone. (F, minority)*
		*Respect for everyone. Embracing our differences and seeing that is what makes us unique. (F, white)*
	Feeling *safe* at work	*Being able to be myself (professionally) at work without judgment of being different. (F, minority)*
		*Ensuring all people feel welcome and safe to work in our department. (M, white)*
	Feeling *heard* at work	*Having a voice and feeling that your voice is heard. (F, white)*
		*Making sure everyone has a voice. (M, white)*
	Open acknowledgment of DEI issues	*Some uncomfortable conversations that are necessary. (F, white)*
		*Being able to talk about diversity issues, so that we can all address them. (F, minority)*
	Being “treated the same”	*It means treating everyone through the same lens of humanity, and ensuring that nobody is treated differently or unjustly based on any aspects of their identity. (M, white)*
	Structural changes	*Not being forced to take PTO on religious holidays I don’t celebrate. (M, white)*
		*Moving away from like hires like. Need to be open to broad concepts of DEI. (N.R.)*
	Concerns about DEI initiatives	*I’m concerned that DEI could mean lowering professionalism trough lowering standards for underrepresented populations. (M, white)*
How will you speak up on DEI issues you encounter?	Direct engagement with involved persons	*Directly talk to the person at first. If needed, talk to supervisor. (F, minority)*
		*I would pull the person aside in person and try to address the issue very quickly so they remember the situation. (F, white)*
	Turning to leadership	*Speaking to a leader that I am comfortable to talk with and counting on he/she to pass the message to higher levels. (M, minority)*
		*I was not being heard, so I finally went to Mayo leadership. It was scary to do it, but I am glad I spoke up. (F, white)*
	Hesitant to speak up because of retaliation or lack of response	*Anonymously. Uncomfortable about approaching those in leadership positions who do not want to acknowledge that they are biased. (M, minority)*
		*I’ve tried in the past, politely and repeatedly, and suffered even more, I don’t feel comfortable trying again. (F, white)*
	Leveraging privilege to boost the voices of others	*In a meeting, when someone is being disregarded, I will affirm their comment and make sure they get the credit for it. (M, white)*
	Be more observant	*For me, speaking up is not the issue. The area I could improve is listening/noticing issues better. (M, white)*
	Giving “benefit of the doubt”	*Starting with an open mind, assume no ill intent, hear the discussion, reframe thoughts, offer suggestions on how to change the communication style, and watch for repeated behaviors. (N.R.)*
		*Ask questions to clarify intent. (F, white)*
What will you do to make HSR more inclusive?	Starting a dialog	*Talk about race, culture and background openly, but respectfully. (F, minority)*
		*Gently and casually discuss what I’m learning about privilege in my personal life with my teammates. (F, white)*
	Mentorship/supporting junior staff	*Continue to recruit and promote young women of color into careers in science and medicine. (F, white)*
		*Use my privilege to highlight others. (M, white)*
	Learning and listening	*Develop open spaces for less-heard voices… then listen and continually educate myself (N.R.)*
	Self-reflection	*Recognize and assess my own behaviors that contribute to exclusion. (F, white)*
		*Self-reflect. Don’t assume that I haven’t been biased or not capable of it. (F, minority)*
	Concrete individual actions	*If helping form a work group, make sure there is diverse representation of individuals included. (M, white)*
		*Get better at pronouncing names correctly. (M, white)*
		*Including preferred pronouns in my email signature. (F, white)*

DEI, diversity, equity, and inclusion; F, female; HSR, health sciences research; M, male; N.R., gender and/or race not reported by respondent. Since poll respondents were asked to positively indicate membership in a given professional or diversity group, the assumption is made that the converse if true for any groups not indicated (e.g., a respondent who did not select “woman or female-presenting” is described as “male” for reporting purposes).

## Discussion

The objective of this formative evaluation was to assess Mayo Clinic Department of HSR faculty and staff perceptions toward individual components of a proposed departmental DEI plan and to explore findings by various diversity and professional subgroups within the department. Overall, HSR faculty and staff conveyed support for the plan; notably, over one-quarter of respondents did not reject any components of the plan. In terms of specific plan activities, the activity most commonly endorsed as important and/or potentially impactful was blinded promotion review processes and activities related to DEI training. In contrast, when asked about less direct approaches – such as subtle “nudges” or reminders built into review and promotion processes about biases in performance feedback that are commonly manifested toward members of diversity groups – respondents expressed less support. Interestingly, attitudes toward some specific plan activities expressed by staff members from certain diversity or professional subgroups diverged from their majority counterparts. It is particularly noteworthy that greater proportions of faculty and staff from racial and ethnic minority groups positively endorsed inclusion nudges and blinded promotion reviews compared to White, non-Latinx staff members, while leaders were also more likely than non-leaders to advocate for annual review nudges.

These findings have several implications for both DEI plan implementation and future work in this area. First, while research suggests that mandatory anti-bias training shows relatively little efficacy in improving diversity and inclusion within organizations [25], training for all HSR faculty and staff, and specifically for leaders, was the most widely endorsed plan activity among respondents in our sample. In addition, while behavioral nudges have demonstrated the greatest impact on mitigating the role of biases in organizational settings, these activities were associated with the least amount of support among our respondents, with the exception of racial and ethnic minority participants. We suggest that, in the context of growing awareness of DEI issues, many department members (especially those who are not from backgrounds historically marginalized in academia or medicine) may default to training as the most rational frontline approach to effecting change. Employees may also wish to give themselves and their colleagues the benefit of the doubt by suggesting that any incidence of bias or discrimination is unintentional and stems from a lack of awareness that can be corrected with training.

Nevertheless, training is a necessary but *not* sufficient scaffold for shaping organizational culture. More concrete approaches, such as behavioral nudges, are needed to overturn and correct ingrained and implicit biases in the system, a fact perhaps recognized more easily by racial and ethnic minority respondents in our sample, who have long been professionally disadvantaged by the impact of these biases. In addition, the concept of “nudges” may be unfamiliar to many audiences, even within an academic setting. Others looking to present on or assess responses to DEI initiatives within their institution should offer specific, concrete examples of “inclusion nudges” to ensure that respondents fully understand the concept. Finally, with regard to the annual review inclusion nudges proposed as an activity in our DEI plan, employees may have felt that an annual “nudge” is insufficient to make substantial or sustained changes to the department’s inclusion climate. In fact, inclusion nudges are currently planned as a quarterly activity at the Mayo Clinic institutional level, an increase in frequency which may impact how employees respond to this idea.

We learned multiple lessons in the process of presenting our DEI plan to HSR department faculty and staff, and we hope that others can utilize these findings to strengthen their own efforts in this space. For instance, others seeking to initiate DEI activities in their institution or department can use this framework as a springboard to assess interest in and attitudes toward specific DEI activities among staff members. It may be particularly useful to contrast differential interest in specific plan components between diverse and nondiverse employees. In addition, based on our experience, we recommend that DEI leaders focus on employee assessments of activities that are *not* already fixed or integral to the plan. In our case, assessment of “belonging” and recruitment of diverse individuals were two non-negotiable plan activities. Our early plan assessment efforts may have yielded more meaningful responses by asking meeting attendees to reflect on the activities about which DEI planners were more ambivalent. Finally, although this exploratory work indicated a high level of DEI plan buy-in from staff members, plan administrators at our institution and others can reasonably expect opposition from those who feel such efforts are unnecessary or even harmful. For example, a small number of respondents in our sample expressed concerns about DEI initiatives “lowering standards” for professionalism or even forcing conformity in attitudes. Although strategies for responding to DEI opposition are not explicitly addressed in our plan, this is an important concern that should be integrated into future DEI initiatives. For example, DEI leaders can proactively defend against similar concerns by explicitly stating that any change to professional or academic standards is not a goal of DEI activities.

The plan described in this special communication has important limitations. Notably, the intended purpose of our plan is to evaluate systematic solutions to help a broad swath of people from different backgrounds, rather than exploring targeted interventions aimed at individuals from specific diversity or professional groups. Our intention is to allow this framework to generalize across many different organizational or departmental contexts, but we recognize that more tailored approaches may be necessary to achieve maximum impact for certain groups or within certain settings.

In addition, the findings reported here also have several methodological limitations, many of which are tied to their intended purpose to inform real-time development of the DEI plan. First, attendees at the HSR all-staff meeting represent only a cross section (approximately 30%) of all HSR employees. Furthermore, poll participation was not mandatory and only a subset of attendees (approximately one-half) responded to these questions during the meeting. As a result, poll respondent perceptions of the DEI plan may not be representative of HSR employees as a whole. However, the distribution of women (59.3% of meeting participants vs. 58.6% of all HSR employees) and underrepresented minority participants (5.6% vs. 5.4% of all employees) were roughly equivalent, increasing our confidence in the generalizability of these findings to the department as a whole. Of note, this low proportion of underrepresented minority departmental members was part of the impetus for this plan. In addition, we conducted multiple comparisons between different groups, potentially limiting the statistical validity of these findings. Again, this was driven by the applied purpose of informing development of the plan within the department, and so we intentionally sought insights from multiple groups on specific plan components. Finally, the predetermined list of personal and professional diversity groups in which poll respondents could self-disclose membership during the poll was not exhaustive and may have excluded the perspectives of other groups, including persons with disabilities, older adults, persons from disadvantaged socioeconomic backgrounds, and others. Based on the authors’ experience, including these groups would be an important consideration for anyone considering this method of prioritizing DEI plan components.

Although the DEI plan described in this report remains in its early stages, we felt a sense of urgency in disseminating the framework, proposed activities, and initial staff reactions to the plan. Racism, sexism, and countless other systemic forms of discrimination against historically marginalized groups have long been pervasive in academic medicine, but recent events (namely, multiple instances of police brutality as well as growing awareness of racial and socioeconomic disparities in morbidity and mortality related to the COVID-19 pandemic) have stressed the importance of addressing racism and discrimination in all parts of US society. Given this prominence on the national agenda, many organizations, including Mayo Clinic [28], have expressly committed to taking action to address and eradicate racism. Therefore, we offer our DEI plan as an early framework for action within specific organizational and departmental contexts, as well as initial staff member reactions to the framework.
